# Role of Liver-Derived Ketones, Hepatokines, and Metabolites in the Regulation of Renal Function

**DOI:** 10.34067/KID.0000000931

**Published:** 2025-07-21

**Authors:** Gertrude Arthur, Michael I. Adenawoola, Sally Wahba, Bentley S. Montgomery, David E. Stec

**Affiliations:** Department of Physiology and Biophysics, Cardiorenal, and Metabolic Diseases Research Center, University of Mississippi Medical Center, Jackson, Mississippi

**Keywords:** renal function, renal injury, signaling, tubular physiology

## Abstract

Alteration in renal function has long been known to be a consequence of liver disease. However, the mechanisms by which the liver can regulate kidney function under basal conditions and in response to liver disease have yet to be fully understood. The liver is a complex organ capable of producing metabolites, including ketones, bile acids, and hepatokines such as fibroblast growth factor 21. Alterations in the hepatic production of these metabolites and hormones can significantly affect renal function and may play a crucial role in the development of kidney disease. The goal of this review is to summarize the mechanisms by which liver-derived metabolites and hepatokines regulate kidney function in health and disease.

## Introduction

The interconnected relationship between the liver and kidney is fully apparent in conditions such as hepatic cirrhosis, which causes alterations in renal blood flow due to portal hypertension and changes in splenic blood flow, resulting in pathologic renal vasoconstriction known as hepatorenal syndrome (HRS).^1^ HRS occurs in two forms: type 1 HRS, which is a rapidly progressing kidney failure, and type 2 HRS, which is a slower, less treatable form often associated with the development of ascites.^2^ Although HRS occurs in the context of advanced liver disease, more subtle changes in liver function can also affect the kidney. For example, evidence is emerging on the effect of increased hepatic steatosis on the kidney. Several population studies have demonstrated an increased risk of CKD in patients with metabolic-associated steatotic liver disease (MASLD).^3–5^ Moreover, the polymorphism in the patatin-like phospholipase domain-containing protein 3 that is responsible for the development of hepatic steatosis in humans has also been linked to the development of CKD in adults and children.^6–8^ These observations demonstrate that the liver influences kidney function without overt signs of liver disease such as advanced cirrhosis.

The liver is a complex organ intimately linked to the metabolism of glucose, lipids, and amino acids. It can also produce endogenous hormones called hepatokines, which can act in both autocrine and paracrine fashions. It is also responsible for synthesizing bile acids, which aid in the digestion of fats in the intestine. Bile acids signal through a family of receptors, including the constitutive androstane receptor, farnesoid X receptor (FXR), pregnane X receptor, and vitamin D3 receptor, to regulate metabolism and inflammation, among other activities. Bile acid receptors are found throughout the entire nephron and regulate salt and water reabsorption through the activation by chenodeoxycholic acid and cholic acid.^9,10^ Thus, the liver plays a crucial role in regulating kidney function under basal conditions, and pathologic changes in liver function can profoundly affect the kidney. This review highlights several key pathways by which the liver can affect kidney function, from alterations in metabolites such as ketone generation to changes in hepatokines that directly affect renal function under basal conditions and in response to subtle changes in liver function associated with hepatic steatosis.

## Liver-Derived Ketones

Hepatic ketogenesis is the primary source of blood ketones, mainly *β*-hydroxybutyrate (BHOB) and acetone. Ketones are produced in the mitochondria of hepatocytes through the metabolism of acetyl-CoA, especially when the levels of oxaloacetate are low and acetyl-CoA cannot enter the citric acid cycle. Ketones can be transported in the blood to organs such as the brain and kidneys, serving as a fuel source or having other modulatory actions. The liver lacks succinyl-CoA transferase, which is essential for processing ketones, thereby limiting its ability to use ketones as an energy source to generate ATP.^11^

BHOB is generated in the liver from acetoacetate by D-*β*-hydroxybutyrate dehydrogenase 1. The main substrate for BHOB generation is acetyl-CoA, generated from the oxidation of fatty acids. BHOB can also be generated from ketogenic amino acids such as leucine, which are metabolized to acetyl-CoA and acetoacetate. D-*β*-hydroxybutyrate dehydrogenase 1 is also expressed in the kidney, and its expression has been reported to be decreased in diabetic kidney disease.^12^ BHOB has been shown to have beneficial and protective effects in kidney diseases, including Alport syndrome, autosomal dominant polycystic kidney disease, and renal ischemia-reperfusion injury.^13–16^ In addition, restoring plasma BHOB levels reduces BP and mitigates renal injury in Dahl salt-sensitive rats fed a high-sodium diet.^17^ There are several potential mechanisms by which BHOB protects the kidney. The first is the modulation of class 1 histone deacetylases (HDACs). HDACs regulate binding of histones to DNA by increasing the positive charge of histone tails by removal of negatively charged acetyl groups. Histone acetylation is a process that plays a crucial role in maintaining fluid and water homeostasis.^18^ Suppression of HDAC activity by BHOB decreases oxidative stress by upregulating antioxidant genes mitochondrial SOD, FoxO3A, and MT2.^19,20^ Superoxide anion is a known regulator of renal vascular and tubular function, and its generation in the kidney plays an important role in regulating BP.^21,22^ Another mechanism by which BHOB protects the kidney is through anti-inflammatory actions. BHOB has been demonstrated to be a potent inhibitor of the NLR family pyrin domain containing 3 inflammasome, modulating the inflammatory FoxO1 and NF-κB pathways through interactions with peroxisome proliferator-activated receptor gamma coactivator 1-alpha.^23–25^ The inhibition of the NLR family pyrin domain containing 3 inflammasome is a significant pathway by which BHOB protects the kidney against cisplatin-induced AKI.^26^ Increased ketogenesis can also affect fatty acid metabolism as ketogenic deficiency has been associated with lower hepatic fatty acid metabolism and increased steatotic liver injury.^27^ Ketones have also recently been reported to support fatty acid elongation and polyunsaturated fatty acid homeostasis in the liver.^28^ A recent study demonstrated that loss of hepatic ketogenesis impaired fatty acid oxidation but did not result in enhancement of metabolic dysfunction-associated steatohepatitis-induced liver injury, suggesting that enhanced ketogenesis protects against lipotoxic injury through pathways distinct from improvements in fatty acid oxidation.^29^ Thus, BHOB may protect the kidney through its regulation of HDACs or anti-inflammatory properties as opposed to its effects on fatty acid metabolism.

**Figure 1:**
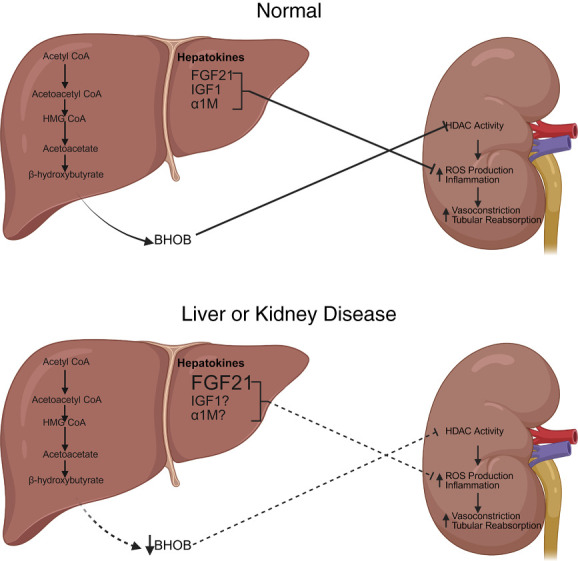
**Top—regulation of kidney function by normal production of BHOB and hepatokines.** BHOB generated from acetyl CoA acts in the kidney to inhibit HDAC activity. HDAC can regulate ROS production and inflammation, affecting kidney function. Hepatokines released from the liver can also affect ROS production and inflammation, thereby regulating kidney function. Bottom—regulation of BHOB and hepatokines in liver or kidney disease. Liver disease can decrease the production of BHOB, decreasing its effects on the kidney, promoting vasoconstriction, and increasing tubular reabsorption. Kidney disease is associated with increased levels of FGF21; however, its effects in the kidney are diminished. The levels of other hepatokines in kidney disease are not well understood. *α*1M, *α*1 microglobulin; BHOB, *β*-hydroxybutyrate; FGF21, fibroblast growth factor 21; HDAC, histone deacetylase; ROS, reactive oxygen species.

Higher levels of plasma BHOB are associated with less decline in GFR in patients with CKD.^14^ However, the mechanisms by which BHOB prevents the decrease in GFR in CKD are not known. Whether BHOB influences HDAC activity to attenuate glomerular and tubular injury or acts in an anti-inflammatory or antioxidant function to preserve renal blood flow (Figure 1) is not known. Interestingly, sodium-glucose cotransporter 2 inhibitors (SGLT2is), which have renoprotective effects in CKD, have been demonstrated to stimulate hepatic BHOB production.^30,31^ Specific studies in which the rise in plasma BHOB is prevented after SGLT2i treatment will clarify the role of increased ketone production in the protective benefits of these inhibitors against the development of CKD.

## Hepatokines

### Fibroblast Growth Factor 21

Fibroblast growth factor 21 (FGF21) belongs to a larger family of fibroblast growth factors (FGFs), comprising of 23 members (FGF1–23), that transduce signaling through the FGF receptors.^32–34^

In the larger family, it is part of a subgroup that requires the binding of a cofactor (*α* or *β*-klotho) for the activation of its receptor.^32,34,35^ This subgroup comprises of FGF15/19, FGF21, and FGF23. Tissues such as the pancreas, white and brown adipose tissue (white adipose tissue [WAT], brown adipose tissue [BAT]), skeletal muscle, heart, and kidney sparsely express FGF21, with the primary source of circulating FGF21 being the liver.^36^ In fact, increased expression of renal FGF21 in mice does not change plasma FGF21.^37^

From the liver, FGF21 is secreted into the plasma, acting as a critical systemic and local regulator of glucose, lipid, and energy metabolism.^38^ Increased circulating levels of FGF21 in rodents improve hyperglycemia, insulin resistance, dyslipidemia, and hepatic steatosis by binding *β*-klotho and activating protein kinase B (Akt) and extracellular signal-regulated kinase 1/2 pathways.^39–42^ In humans, FGF21 treatment improves dyslipidemia by increasing high-density lipoproteins and decreasing triglyceride levels.^43–46^ The effects of FGF21 on glucose and insulin balance are controversial, with studies in rodents demonstrating that FGF21 regulates glucose and insulin levels; however, studies in humans have failed to find such effects. In rodents, FGF21 regulates glucose and insulin balance in the pancreas, WAT, liver, and skeletal muscle, as well as lipolysis in WAT, BAT, and the liver. In addition, it influences the circadian rhythm and sympathetic nervous system in the brain of rodents.^32,36^ FGF21 also reduces oxidative stress and inflammation in the kidney and heart and prevents renal lipid accumulation.^32,36^ The function of FGF21, particularly in WAT, to regulate glucose, lipid, and energy metabolism is important because these factors influence the adipose-kidney axis and determine how certain adipokines, such as adiponectin and leptin, affect kidney function.^47^ Moreover, the autocrine function of FGF21 on the liver mediates hepatic lipid metabolism and the production and release of other hepatokines that are important for regulating kidney function.^32,33,36^

In the kidney, FGF21 reduces oxidative stress by augmenting the autophagic process. In both young and old mice, loss of FGF21 results in damaged cellular lysosomes, leading to increased flux in autophagy and autophagy burden.^37^ FGF21, signaling through the Wnt/*β*-catenin pathway, also reduces renal fibrosis and inflammation. In CKD mouse model of unilateral ureteral obstruction, increased FGF21 inhibited the Wnt/*β*-catenin pathway, alleviating renal fibrosis.^48^ Inflammatory markers, IL-1*β* and TNF-*α*, were also reduced by increasing renal FGF21 expression.^48^ Several reports indicate the benefit of increased circulating or renal FGF21 levels in diabetic nephropathy (DN). In diabetic *db/db* mice, daily doses of FGF21 markedly improved insulin resistance and glomerular morphologic abnormalities, leading to decreased urinary albumin excretion and mesangial expansion.^49^ FGF21 also ameliorates DN by improving autophagy through the 5' AMP-activated protein kinase/mammalian target of rapamycin pathway in a leptin receptor knockout (KO) model of diabetes.^50^ In mice treated with streptozotocin to induce type 1 diabetes, fenofibrate treatment induced an FGF21-dependent renoprotection by activating the Akt2/GSK-3*β*/Fyn/Nrf2 and 5' AMP-activated protein kinase pathway.^51,52^ *In vitro*, increased expression of FGF21 in human mesangial cells incubated with high glucose showed decreased collagen 4 and fibronectin levels through STAT5 signaling pathway.^53^ Diabetic medications such as metformin and SGLT2i increase the expression of renal FGF21, suggesting its importance in the signaling pathway that these medications use to improve kidney function.^54–56^

Paradoxically, FGF21 is increased in renal pathologic conditions such as DN and CKD compared with healthy controls in humans (Figure 1). Elevated plasma FGF21 was a predictor of GFR decline and CKD progression in patients with type 2 diabetes.^57^ A comparison of plasma FGF21 in kidney transplant patients before and after successful transplantation showed a dramatic decrease in plasma FGF21 after kidney transplantation.^58^ FGF21 levels are also independently associated with urinary albumin excretion and are elevated in DN patients with microalbuminuria compared with those without.^59^ It has been speculated that elevated FGF21 in DN and CKD is due to tissue resistance similar to insulin resistance.^60^ Moreover, comorbid conditions that often accompany DN and CKD, such as obesity, dyslipidemia, diabetes, MASLD, and hypertension, exhibit elevated circulating FGF21 levels (Figure 1), all of which may contribute to increased resistance to the beneficial effects of FGF21 in the kidney.^32,60^ However, Nakano *et al.* demonstrated that elevated plasma FGF21 occurs as a survival response to CKD at the expense of increased BP in mice.^61^

### IGF-1

Most growth hormone (GH) effects on the kidneys are mediated by IGF-1, which is mainly secreted from the liver. The secretion of IGF-1 in the liver is controlled by GH acting through the Janus kinase 2 signaling pathway.^62^ The degree of hepatic steatosis is inversely correlated with the level of hepatic IGF-1 expression.^63^ The same correlation was found between nonalcoholic steatohepatitis (NASH) activity score, body mass index, and IGF-1 expression levels. Hence, treatment with a GH-releasing hormone agonist increases IGF-1 levels, thereby decreasing NASH activity score and hepatocyte ballooning without altering steatosis.^63^ On the other hand, IGF binding protein-7 was shown to be directly correlated with the degree of steatosis and fibrosis in NASH.^63^ A study in a GH-deficient and hepatic IGF-1-deficient mouse model showed increased hepatic triglycerides after GH treatment.^62^ These findings suggest that IGF-1 plays a role in liver adiposity along with GH itself.

IGF-1 exerts its effects on the kidney through binding to IGF-1 receptor that is found in both renal glomeruli and tubules. Physiologically, IGF-1 causes vasodilation of afferent and efferent arterioles, which increases renal blood flow and GFR.^64^ It is known that IGF-1 increases GFR in humans by 25% by acutely stimulating nitric oxide synthesis in these vessels and chronically through renal hypertrophy.^64^ In renal tubules, IGF-1 enhances the sodium-phosphate cotransporter, increasing phosphate reabsorption in the proximal convoluted tubules (PCTs) and also indirectly increases calcium reabsorption through the stimulation of 1-*α*-hydroxylase, which in turn increases active vitamin D.^65^ More recently, IGF-1 has been found to regulate the expression and post-translational modifications of human organic anion transporters, a family of cell membrane proteins located in PCTs, which are responsible for the renal handling of drugs, toxins, and metabolites.^66^ The distal nephron segments increase sodium and water reabsorption by epithelial sodium channels.^65^ In hepatic IGF-1 knock-out mice, despite high GH levels, IGF-1 gene KO mice had smaller kidneys, increased urine volume, and urinary sodium content.^67^ These findings support the hypothesis that these effects are due to IGF-1 and not the direct action of GH on the kidneys. Hepatic IGF-1 deficiency was also found to elevate systolic BP in desoxycorticosterone acetate-salt hypertension mice compared with mice with normal IGF-1 levels.^68^ To differentiate the role of liver IGF-1 from IGF-1 produced locally in the kidney due to high GH in the hepatic IGF-1 KO mice, Nordstrom *et al.* developed a mouse model that is unable to secrete GH from the pituitary and is deficient in IGF-1 in the liver. They found that these mice had smaller kidney size with or without external GH treatment. These findings suggest that liver-derived IGF-1, not IGF locally produced in the kidney, is important in regulating kidney function.^62^

IGF-1 has also been reported to increase GFR and improve PCT survival in mouse models of AKI.^65^ Human clinical trials of IGF-1 were found to increase GFR in ESKD. Moreover, patients with ESKD treated with intermittent low-dose recombinant IGF-1 had elevated inulin clearance, higher than that achieved with dialysis alone and more than that of the placebo-treated patients.^69^ However, more attention has been given to IGF-binding proteins (IGFBPs) since they were found to have biologic effects on their own other than regulating the bioavailability of IGF-1.^70^ IGFBP-2 was found to be a sensitive marker for the severity of nephrotic syndrome in children. At the same time, in adults, the degree of proteinuria and BUN, markers of renal injury, was better correlated with acid labile substance, the third compound in the IGF-1-IGFBP-acid labile substance ternary complex.^64^ These results suggest that different parts of the IGF-1 ternary complex can serve as markers of renal injury in various patient populations.

### *α*1 Microglobulin

Although widely expressed in various tissues, *α*1 microglobulin (*α*1M) is primarily produced in the liver.^71–75^ On secretion into the bloodstream, through a reduction-resistant disulfide link, about 50% of plasma *α*1M combines with monomeric IgA to create a 1:1 complex in human plasma.^76^ Albumin and prothrombin also create complexes with *α*1M at a ratio of 7% and 1%.^76^ *α*1M functions as a physiologic antioxidant and protects against the propagation of *α*-particle irradiation-induced damage by suppressing cell death, apoptosis, oxidative stress, and damage in cells.^77,78^ Coadministration of *α*1M with 177 Lutetium-DOTA-(Tyr3)-octreotate, a radiolabeled somatostatin analog, produced tissue-specific proteomic responses, especially in the bone marrow and kidney. *α*1M, along with haptoglobin, is essential for protecting against the harmful effects of hemoglobin-induced AKI. Both *α*1M and haptoglobin attach to free hemoglobin and heme (which can build up in the kidneys during hemolysis) to prevent renal tubule apoptosis and damage.^79–81^ *In vitro*, using human proximal tubule epithelial cells, Kristiansson *et al.* demonstrated that *α*1M binds heme at a 2:1 ratio and protects from heme-induced tubule cell damage.^82^ *α*1M also acts as a free radical scavenger in kidney tubules.^83^ The 2,2′-azino-bis (3-ethylbenzothiazoline-6-sulfonic acid) radical is reduced by *α*1M by forming covalent bonds with the radical and could potentially serve as a scavenger *in vivo*.^83,84^ The *t*_1/2_ of *α*1M in blood is believed to be roughly 1–3 minutes,^85^ and most free *α*1M is reabsorbed by proximal tubule cells and catabolized into the urine after it is nearly freely filtered past the glomerular membranes because of its small size.^86^ However, some *α*1M is excreted in the urine, which could be a useful clinical marker for renal tubular injury.^86^ Excessive excretion of *α*1M is considered a sensitive indicator of impaired renal tubular function^87^ and a marker of the severity of CKD progression.^88^

## Bilirubin Metabolism by the Liver

**Figure 2:**
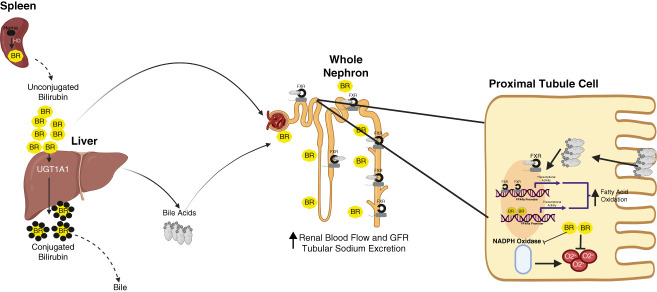
**Schematic of the effect of BR and bile acids on the whole kidney and proximal tubule cells.** BR is derived from the spleen through the metabolism of heme by HO. It travels in the blood to the liver, where it gets conjugated by UGT1A1 for elimination in the bile. Unconjugated BR can also travel in the blood to the kidney, where it increases renal blood flow and promotes sodium and water excretion. Bile acids are also produced by the liver and act by the FXR located throughout the nephron to affect kidney function. In the proximal tubule, BR binds to the promoter of the PPAR*α* to increase transcription of genes that increase fatty acid oxidation. In addition, BR also acts as an antioxidant, inhibiting NADPH oxidase and directly scavenging superoxide anions. FXR is activated by the reabsorption of bile acids from the apical membrane to activate the PPAR*γ* to increase fatty acid oxidation. BR, bilirubin; FXR, farnesoid X receptor; HO, heme oxygenase; PPAR*α*, peroxisome proliferator-activated receptor *α*; PPAR*γ*, peroxisome proliferator-activated receptor *γ*; UGT1A1, UDP-glucuronosyltransferase 1.

Plasma bilirubin (BR) levels are believed to be mainly derived from the breakdown of red blood cells in the spleen and to a small extent liver and bone marrow^89^ (Figure 2). BR generated from the breakdown of heme released by red blood cells travels in the blood bound to albumin where it enters the liver. In the liver, BR is conjugated by the hepatic UDP-glucuronosyltransferase 1A1 (UGT1A1), which is then excreted into the bile for elimination through the gut. Mutations in hepatic UGT1A1 decrease the conjugation of BR, which increases the unconjugated levels of BR in the plasma. Different mutations in hepatic UGT1A1 can lead to severe hyperbilirubinemia, as seen in Crigler-Najjar syndrome, or more physiologic elevations in plasma BR, as observed in patients with Gilbert syndrome. Increased plasma BR levels have been linked to protection from CKD, diabetes-induced nephropathy, and ischemia-induced injury.^90–94^ Alterations in hepatic UGT1A1 levels, observed in hyberbilirubinemic Gunn rats with a genetic mutation in UGTA1 or through targeting by antisense morpholinos, increase serum BR levels and protect against hypertension, while preserving renal blood flow and GFR in response to pressor doses of angiotensin II.^95–97^ BR is a potent endogenous antioxidant that is capable of scavenging reactive oxygen species as well as inhibiting NADPH oxidase, a major source of superoxide anion production.^98,99^ However, the effects of moderate hyperbilirubinemia on BP are not fully accounted for by the antioxidant properties of BR.^100^ Recently, BR has been demonstrated to be a signaling molecule capable of activating the transcription factor peroxisome proliferator-activated receptor *α* (PPAR*α*)^101–103^ (Figure 2). Although the activation of renal PPAR*α* with fibrates has been demonstrated to lower BP and promote natriuresis,^40–42^ the importance of renal PPAR*α* activation in the antihypertensive actions of BR remains to be determined.

## Bile Acids

Bile acids are produced by hepatocytes in the liver, where they mainly aid in the reabsorption of lipids in the intestine. However, they also circulate in the blood and act through a series of receptors to modulate cell and tissue function. The FXR is a bile acid receptor found throughout the nephron (Figure 2). Several studies have demonstrated that FXR levels are decreased in the kidneys of individuals with diabetes.^104^ Treatment with FXR agonists improves lipid metabolism, reduces oxidative stress, proteinuria, glomerulosclerosis, and fibrosis in several models of DN.^105–107^ These studies suggest a potentially beneficial role for bile acids and the activation of bile acid receptors in preventing kidney injury in response to diabetes and aging. This view is further supported by a recent study by Geng *et al.*, which demonstrates that a higher concentration of primary bile acids in the plasma is associated with lower odds of developing CKD in patients with type 2 diabetes.^108^ These findings are in concert with the results of another population study, which reported that lower levels of bile acids in plasma were an independent risk factor for developing ESKD in patients with type 2 diabetes.^109^ Although these studies suggest a protective role for bile acids, there are situations in the kidney where increased bile acids may promote renal injury. Cholemic nephropathy is a condition in which excess bile acid levels, either through liver disease or biliary obstruction, result in the development of AKI. The proximal tubule reabsorbs most filtered bile acids, and enhanced reabsorption of bile acids can promote oxidative damage, inflammation, and the release of vasoactive substances, leading to increased renal vasoconstriction and decreased renal function.^110^ The FXR can also activate peroxisome proliferator-activated receptor *γ*, increasing fatty acid oxidation, which may be protective against AKI and CKD (Figure 2). However, the role of bile acids in promoting renal injury during cholestasis is very controversial. It has been argued that the bile casts observed in these patients could be secondary phenomena resulting from other factors, such as diminished washout of casts due to reduced kidney function. Further clinical and preclinical studies are needed to resolve this controversy.

## Conclusions

This review has highlighted several pathways by which the liver can affect kidney function in health and disease. However, a deeper physiologic understanding of the role of liver-derived metabolites and hepatokines is needed. For example, the complex role of FGF21 in CKD, where FGF21 levels seem to be elevated early on and then decline with the progression of CKD. The kidney exhibits resistance to the effects of FGF21 early on in CKD; however, further studies in preclinical models of CKD are needed to determine the mechanism by which FGF21 seems to lose its protective actions on kidney function in the early stages of CKD. BHOB has been shown to lower BP and reduce renal inflammation; however, the mechanism by which it acts in the kidney is not fully understood. Several clinical studies have demonstrated the beneficial actions of ketogenic diets on the progression of CKD and autosomal dominant polycystic kidney disease^111,112^; however, the effects of ketogenic diets on the development of CKD in MASLD patients remain to be tested. Plasma BR levels are known to be decreased in CKD.^113^ Clinical trials are needed in which plasma BR levels are increased through the inhibition of hepatic UGT1A1 or the use of BR nanoparticles (136) to determine the effectiveness of these approaches in combating the progression of CKD.

As the intricate relationship between the liver and the kidney is further understood, several components need to be developed so that the discoveries in this area can be translated into benefits for patient populations. As the incidence of MASLD increases, complications such as cardiovascular disease (CVD) and CKD are also rising in this patient population. The role of altered hepatokines and metabolites in contributing to this increased susceptibility requires further examination. For example, while FXR receptor agonists have been approved or are in phase 2 and 3 clinical trials for metabolic dysfunction-associated steatohepatitis,^114^ only one clinical trial has been conducted in DN, and no trials have been undertaken to assess the effectiveness of FXR agonists for CKD. The potential therapeutic role for FXR receptor agonists in MASLD-induced CVD and CKD also needs to be evaluated through patient population studies and clinical trials. The emergence of therapies such as SGLT2i and glucagon-like peptide-1 receptor agonists offers promise for the treatment of MASLD; however, their effects on hepatic metabolites and hepatokines remain to be determined. Both preclinical and clinical studies on the effectiveness of this new class of drugs in the setting of MASLD-induced CVD and CKD are warranted.
